# Preclinical Evaluation of a Noncontact Simultaneous Monitoring Method for Respiration and Carotid Pulsation Using Impulse-Radio Ultra-Wideband Radar

**DOI:** 10.1038/s41598-019-48386-9

**Published:** 2019-08-15

**Authors:** Jun-Young Park, Yonggu Lee, Yeon-Woo Choi, Ran Heo, Hyun-Kyung Park, Seok-Hyun Cho, Sung Ho Cho, Young-Hyo Lim

**Affiliations:** 1 Department of Electronics and Computer Engineering, Hanyang University, Seoul, 04763 Republic of Korea; 2 Division of Cardiology, Department of Internal Medicine, College of Medicine, Hanyang University, Seoul, 04763 Republic of Korea; 3 Department of Pediatrics, College of Medicine, Hanyang University, Seoul, 04763 Republic of Korea; 4 Department of Otorhinolaryngology, College of Medicine, Hanyang University, Seoul, 04763 Republic of Korea

**Keywords:** Cardiovascular biology, Medical imaging, Biomedical engineering

## Abstract

There has been the possibility for respiration and carotid pulsation to be simultaneously monitored from a distance using impulse-radio ultra-wideband (IR-UWB) radar. Therefore, we investigated the validity of simultaneous respiratory rates (RR), pulse rates (PR) and R-R interval measurement using IR-UWB radar. We included 19 patients with a normal sinus rhythm (NSR) and 14 patients with persistent atrial fibrillation (PeAF). The RR, PR, R-R interval and rhythm were obtained simultaneously from the right carotid artery area in a supine position and under normal breathing conditions using IR-UWB radar. There was excellent agreement between the RR obtained by IR-UWB radar and that manually counted by a physician (intraclass correlation coefficient [ICC] 0.852). In the NSR group, there was excellent agreement between the PR (ICC 0.985), average R-R interval (ICC 0.999), and individual R-R interval (ICC 0.910) measured by IR-UWB radar and electrocardiography. In the PeAF group, PR (ICC 0.930), average R-R interval (ICC 0.957) and individual R-R interval (ICC 0.701) also agreed well between the two methods. These results demonstrate that IR-UWB radar can simultaneously monitor respiration, carotid pulse and heart rhythm with high precision and may thus be utilized as a noncontact continuous vital sign monitoring in clinical practice.

## Introduction

Continuous vital sign monitoring is one of the most fundamental components of patient care in numerous medical situations, including emergency departments, critical care units and peri-procedural states. Further, continuous vital sign monitoring may also prevent sudden death in elderly patients and infants^1–4^. Conventional vital sign monitoring methods, including electrocardiography (ECG), pulse oximetry and impedance pneumography, typically require adhesive transcutaneous sensors attached to multiple wires, which may limit the mobility of patients and cause discomfort, skin injuries and even contagious infections^4–6^. Even wearable devices that have recently been developed for vital sign monitoring cannot avoid these issues. However, noncontact vital sign monitoring methods may provide a solution.

Radar detects and traces the movement of an object by transmitting radio waves of large bandwidth and receiving the waves reflected by the object. Because impulse-radio ultra-wideband (IR-UWB) radar occupies a wide frequency band of >20% of the fractional bandwidth, its wave transmission can be delivered as a form of impulses in the time domain, which gives IR-UWB radar many advantages, including good penetration power, high multipath resistance, high range resolution and simple hardware structure^7^. These advantages enable IR-UWB radar to detect respiration and heart beat from a distance^8,9^.

Since Thiel *et al*. reported the possibility of detecting myocardial motion using UWB radar during breath-holding^10^, many researchers have introduced various measurement methods and data processing algorithms for noncontact vital sign monitoring using UWB radar. Recent studies have shown the possibilities of measuring the heart rate (HR) using radar in subjects during respiration^11,12^, in moving subjects^13^ and in subjects behind a wall^14^. Shi *et al*. have even reported that carotid pulse waveforms could be measured in a noncontact manner, using continuous-wave radar^15^. Despite these technical improvements in vital sign monitoring using radar, accurate recognition of individual heartbeats in the midst of overwhelming respiratory activity remains challenging. Moreover, to date, few studies have reported the performance or validity of their vital sign monitoring methodologies using radar in clinical settings. Studies have also seldom focused on measurement of R-R interval^16,17^, which would have important prospects in cardiovascular researches, such as in automatic arrhythmia detection and HR variability.

We recently reported that IR-UWB radar can accurately measure the HR and R-R interval and can even precisely differentiate the types of heart rhythms of healthy volunteers with normal sinus rhythm (NSR) and patients with persistent atrial fibrillation (PeAF)^17^. However, in the previous study, in which the radar measurements were taken from the anterior chest, it was difficult to isolate the heartbeat signals in the presence of respiratory activity. Therefore, the participants were asked to hold their breath for a short period during the radar measurements to exclude signals from respiratory activity, which would hardly be practical for a continuous vital sign monitoring.

To overcome this limitation, we modified the previous noncontact method using IR-UWB radar to monitor the respiratory rate (RR), pulse rate (PR), R-R intervals and heart rhythms continuously from the patient’s neck. In this study, we investigated the validity of the new continuous noncontact vital sign monitoring method by comparing measurements from cardiovascular disease patients obtained using IR-UWB with measurements obtained through conventional methods.

## Subjects and Methods

### Patients

Patients who visited the outpatient clinic of the Department of Cardiology in Hanyang University Medical Center with documented cardiovascular diseases were voluntarily enrolled in this study. We recruited similar numbers of patients with either NSR or PeAF. Patients with debilitating conditions, undiagnosed illnesses or severe or uncontrolled symptoms of cardiovascular diseases were excluded. All patients provided information for demographics, anthropometries and past medical histories and underwent standard 12-lead ECG before the radar measurements were obtained. Written informed consent was obtained from all patients before they were enrolled in the study. The Institutional Review Board of Hanyang University Hospital reviewed and approved the study protocols and monitored the study processes. All methods were performed in accordance with relevant guidelines and regulations (IRB No. 2017-05-004-001).

### Experimental environment and data acquisition

**Figure 1:**
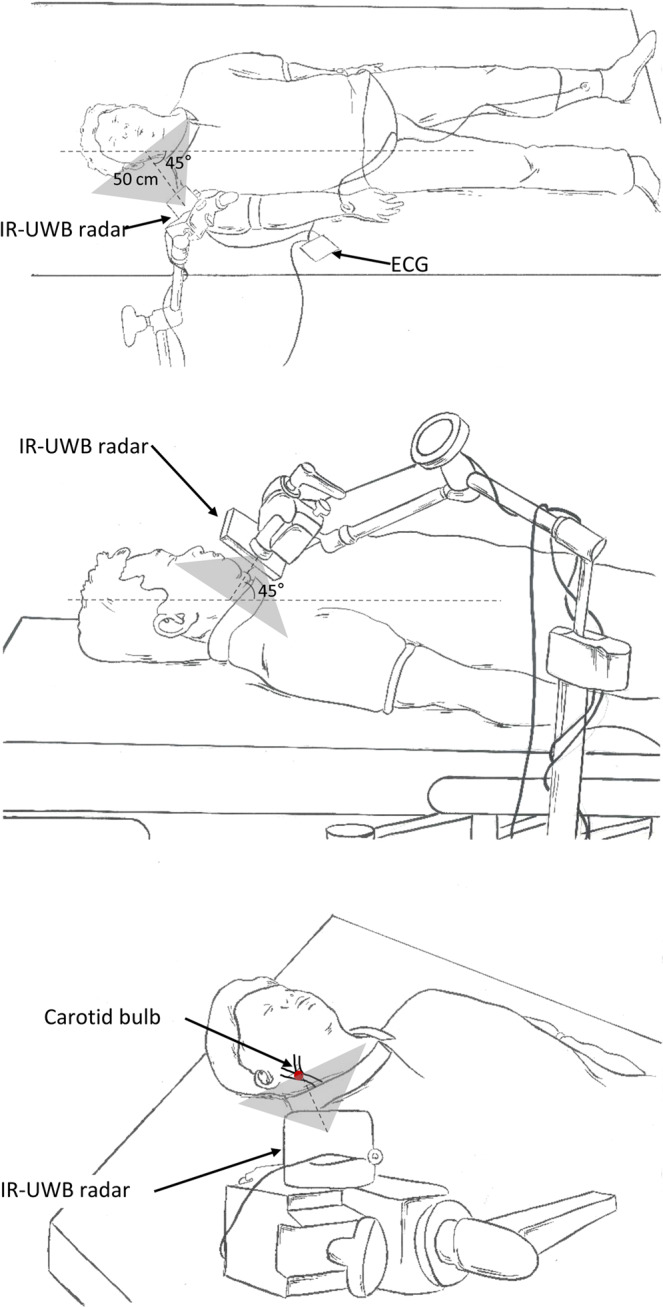
Simplified description of the experimental setting. IR-UWB radar and ECG measurements were obtained simultaneously from patients in a supine position breathing normally after a 5-minute rest. All patients were fully clothed and were asked not to move the upper part of their bodies during the measurements. The radar transmitter was placed at a 50-cm distance from the point 2 cm below the right mandibular angle and at a 45-degree angle with the bed on all three body planes, to aim at the right carotid bulb (red dot). Single lead ECG (lead II) was measured with 3 electrodes placed on the wrists and the right ankle.

The measurements were conducted in a consultation room located in the outpatient clinic of the Cardiology Department of Hanyang University Hospital. The experimental environment is depicted in Fig. 1. Measurements were obtained from patients in a supine position and under normal breathing conditions after 5-minutes’ rest on a bed. The patients were fully dressed and breathing naturally but were asked to minimize their head and neck movement during the measurements. The IR-UWB radar sensor was placed at a 50-cm distance from the point 2 cm caudal to the right mandibular angle at a 45-degree angle with the bed on all three body planes to aim at the carotid bulb, as described in Fig. 1. From this unique angle and position, we could detect signals from movements related to carotid artery pulsation using the IR-UWB radar while obtaining markedly reduced but still recognizable signals from respiratory activities in normal breathing conditions. The radar measurements were collected for 1 minute for each patient, simultaneously with manual counting of the RR by a physician and ECG recording for the HR, R-R interval and rhythm analysis. A commercially available device (XK300-VSA, Xandar Kardian, Delaware, USA) was used for the IR-UWB radar measurements. MATLAB (MathWorks, New York, MA, USA) was used to collect, store and process the raw radar and ECG signal input. Detailed specifications of the radar device are summarized in Supplementary Table 1.

### RR and ECG measurement

ECG was recorded simultaneously with the radar measurements using a single bipolar lead device (PSL-iECG2, PhysioLab, Pusan, Republic of Korea). Three electrodes were attached to the patient’s wrists and right leg, and the data from limb lead II were recorded. The R peaks in the ECG records were automatically recognized using our own algorithms.

### Processing of the IR-UWB radar data

The electromagnetic impulse waves transmitted from the IR-UWB radar and received from the subject’s neck area were digitized and sent to a laptop computer, where the received signals were processed using designated algorithms that we developed in MATLAB to remove background clutter signals as previously described^17^. During experiments, researcher who operated the radar sensor and the laptop computer processing the raw radar data were blinded to the RR and ECG waveform.

**Figure 2:**
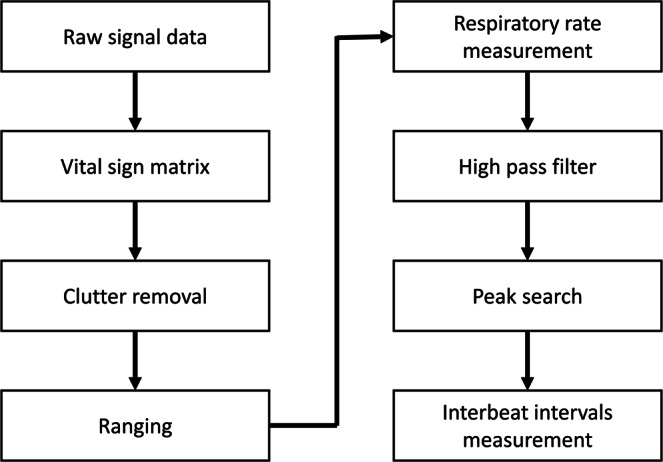
Data processing flow. The radar sensor accumulated the received raw signals and generated a vital sign matrix for the measured period. Background data, also called clutter, were subtracted from the raw signal so that the target with respiratory motion could be detected. After ranging the target, the vital signal was obtained from the radar signal, including the respiratory and carotid pulse signals. The respiratory rate was estimated from the fast Fourier transform of the vital signal, and only the carotid pulse signal remained after passing the signal through a high-pass filter. From the carotid pulse signal, the peaks were identified and interbeat intervals were measured from the peak intervals.

The target signal location was determined as previously described^9^. The IR-UWB radar system that we employed quantizes 1 meter into 156 bins. After the clutter removal, the target location was determined by the SD of each bin’s variation, and carotid pulsation and respiratory activity were estimated through localization of the target. Through fast Fourier transform, the RR was estimated by recognition of the largest signals^9^. The signals from the carotid pulses were obtained by removing the signals from the respiratory activity using a high-pass filter. Because the radar signals still contained unwanted noise, such as thermal noise, after the high-pass filter was applied, a low-pass filter was applied to the radar signals to reduce the noise and allow the heartbeat recognition algorithm to operate. The signal processing steps are summarized in Fig. 2.

**Figure 3:**
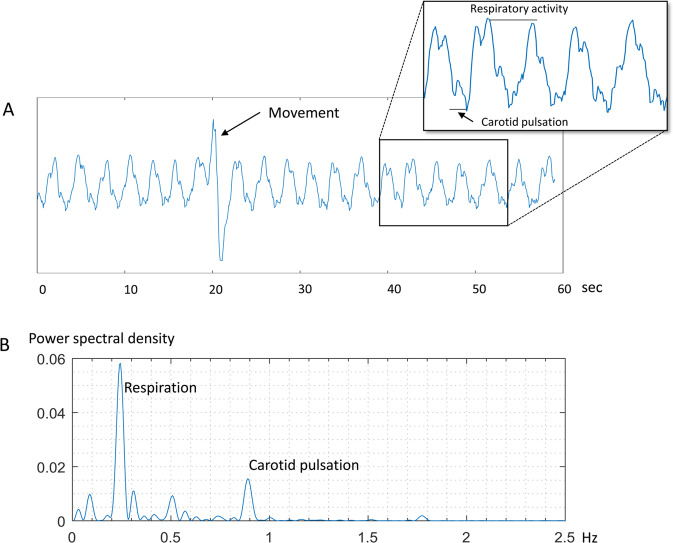
Examples of signals measured using IR-UWB radar. The radar signals mainly consisted of cyclic oscillations generated by respiratory activity. Each time the subject moved, a large fluctuation occurred in the signal (**A**). In the power density spectrum, the signals from respiratory activity occupied the most prominent frequency, approximately between 0.25 Hz and 0.5 Hz, and the signals from carotid pulses occupied the second most prominent frequency, at approximately 0.89 Hz, as its harmonic component (**B**). The power of the respiratory activity signal was approximately 6.3 dB larger than that of the carotid pulsation signal.

Radar signals from a moving subject contained wild fluctuations, whereas signals from a stationary subject mainly consisted of movement signals from respiratory activity and carotid pulsation (Fig. 3A). The respiratory activity showed the most prominent signal power, which was approximately 6.3 dB larger than the second most prominent signal power of the carotid pulses (Fig. 3B). Through automatic recognition of the numbers of peaks and the intervals between the peaks in the heartbeat signals by our own algorithm, the PR, R-R interval and the rhythm were estimated.

### PeAF detection using the frequency domain of the radar signals

Because the R-R interval in patients with PeAF randomly varies over time, PeAF could be distinguished by recognizing the amount of change in the main frequency component^17^. The frequencies obtained 20 times per second were averaged in a 3-second window, and the signal intensities of the frequencies were color-coded and plotted against time for 17 seconds in a 2-dimensional spectrograph of the power spectral density. The frequency variation in the peak signal intensity was calculated for each measurement, and the presence of PeAF was determined using the maximum frequency variation in the peak signal intensity (MaxΔfrq).

### Statistical analysis

Baseline data including age, height, weight, body mass index, blood pressure, RR, HR and R-R interval, are presented as the mean ± SD, and categorical data including sex and past medical histories were presented as the number (%). The agreement of the RR, PR, average R-R interval and individual R-R interval measured by radar with those measured by manual counting and ECG was evaluated using the intraclass correlation coefficient R (ICCR). Bland-Altman (BA) plots were used to graphically present the amount of bias between the radar measurements and the conventional measurements. The cut-off value for the MaxΔfrq was decided using Youden’s J index in the receiver operating characteristics curve analysis. The diagnostic performance of radar for PeAF detection was evaluated against the rhythm detected by ECG. Cohen’s Kappa test was performed to evaluate the level of agreement in PeAF detection between radar and ECG. All statistical analyses were performed using the statistical software R version 3.4.0 and its packages ICC, MethComp, descr and psych. A *p*-value < 0.05 was considered significant.

## Results

A total of 35 patients (23 with NSR and 21 with AF) were recruited. The clinical characteristics and inclusion results of the screened patients are summarized in Supplementary Table 2. Among them, 2 patients (1 with NSR and 1 with AF) were excluded because premature ventricular contractions were frequently present and 33 patients (19 in the NSR group and 14 in the PeAF group) were included in the statistical analyses. The baseline characteristics of the patients are summarized in Table 1. The mean HR was 69.0 ± 3.0 beats/min in the NSR group and 80.0 ± 5.6 beats/min in the PeAF group. The most frequent underlying diseases were coronary artery disease in the NSR group and hypertension in the PeAF group.

**Table 1:** Baseline characteristics of the two groups of participants.

	NSR group	PeAF group
	N = 19	N = 14
Age (year)	62.6 ± 12.9	68.8 ± 11.9
Sex (male)	17 (89.4%)	8 (57.1%)
Height (cm)	164.6 ± 7.6	164.1 ± 12.2
Weight (kg)	69.7 ± 10.5	63.7 ± 16.2
Body mass index (kg/m²)	25.8 ± 3.9	22.0 ± 4.9
Systolic blood pressure (mmHg)	123.0 ± 15.0	120.0 ± 12.8
Diastolic blood pressure (mmHg)	74.7 ± 11.3	78.5 ± 9.9
Heart rate (beats/minute)	69.0 ± 13.0	80.0 ± 5.6
Underlying diseases		
Hypertension	7 (36.8%)	6 (42.8%)
Diabetes	4 (21.0%)	2 (14.2%)
Coronary artery diseases	8 (42.1%)	1 (7.1%)
Congestive heart failure	0 (0.0%)	4 (28.5%)
Cerebrovascular disease	3 (15.7%)	2 (14.2%)
Chronic kidney disease	0 (0.0%)	1 (7.1%)

Data are described as the mean ± SD or N(%).

**Figure 4:**
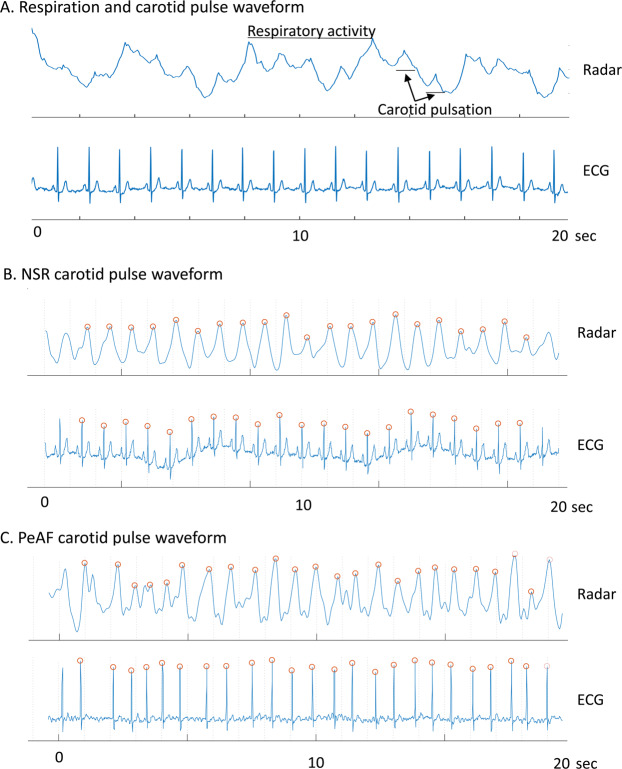
Representative radar signal waveforms of respiratory activity and the carotid pulses. After the removal of clutter, the signal waveform was composed of large cyclic waves from respiratory activity and small but recognizable peaks from carotid pulsation (**A**). After high- and low-pass filters were applied, and the waveform was smoothed over 3 data points, the peak points on the carotid radar pulse waveform appeared to be well-correlated with R waves on ECG in both patients with NSR (**B**) and those with PeAF (**C**). The red circles at each peak in the radar waveform indicate the systolic phase of the carotid pulsation corresponding to each ECG R wave.

The representative signal waveforms measured using IR-UWB radar and passed through the data processing algorithms are depicted in Fig. 3. The signal waveforms were mainly composed of the signals from respiratory activity, and the signals from carotid pulses were recognizable as relatively small peaks within the much larger troughs and crests of the waveforms (Fig. 4A). After the signal processing algorithms were applied, the signals from respiratory activity were removed, and those from carotid pulses remained in the waveforms. The locations of the peaks and the intervals between the neighboring peaks shown in the carotid pulse waveforms appeared to be highly correlated to the R waves and R-R intervals in the ECG data, respectively, in both patients with NSR and those with PeAF (Fig. 4B,C).

**Figure 5:**
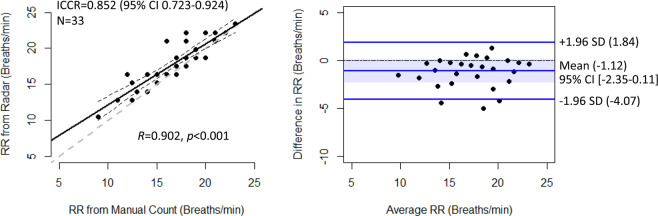
Agreement between the RRs measured by manual counting and those measured using the radar. RRs measured using radar were highly correlated with RRs measured by manual counting, and the BA plot shows no significant difference between the two RR measurement methods. ^†^ICCR, intraclass correlation coefficient; SD, standard deviation.

The levels of agreement between the vital sign measurements obtained using IR-UWB radar and conventional methods were assessed using ICCR and BA plot. The RRs measured using IR-UWB radar agreed well with those measured by manual counting (ICCR 0.852; 95% confidence interval [CI], 0.723–0.924; Fig. 5). The BA plot showed no bias between the RRs measured using the radar and those counted manually, and the numerical difference between the RRs measured using the two methods was only approximately 1.12.

**Figure 6:**
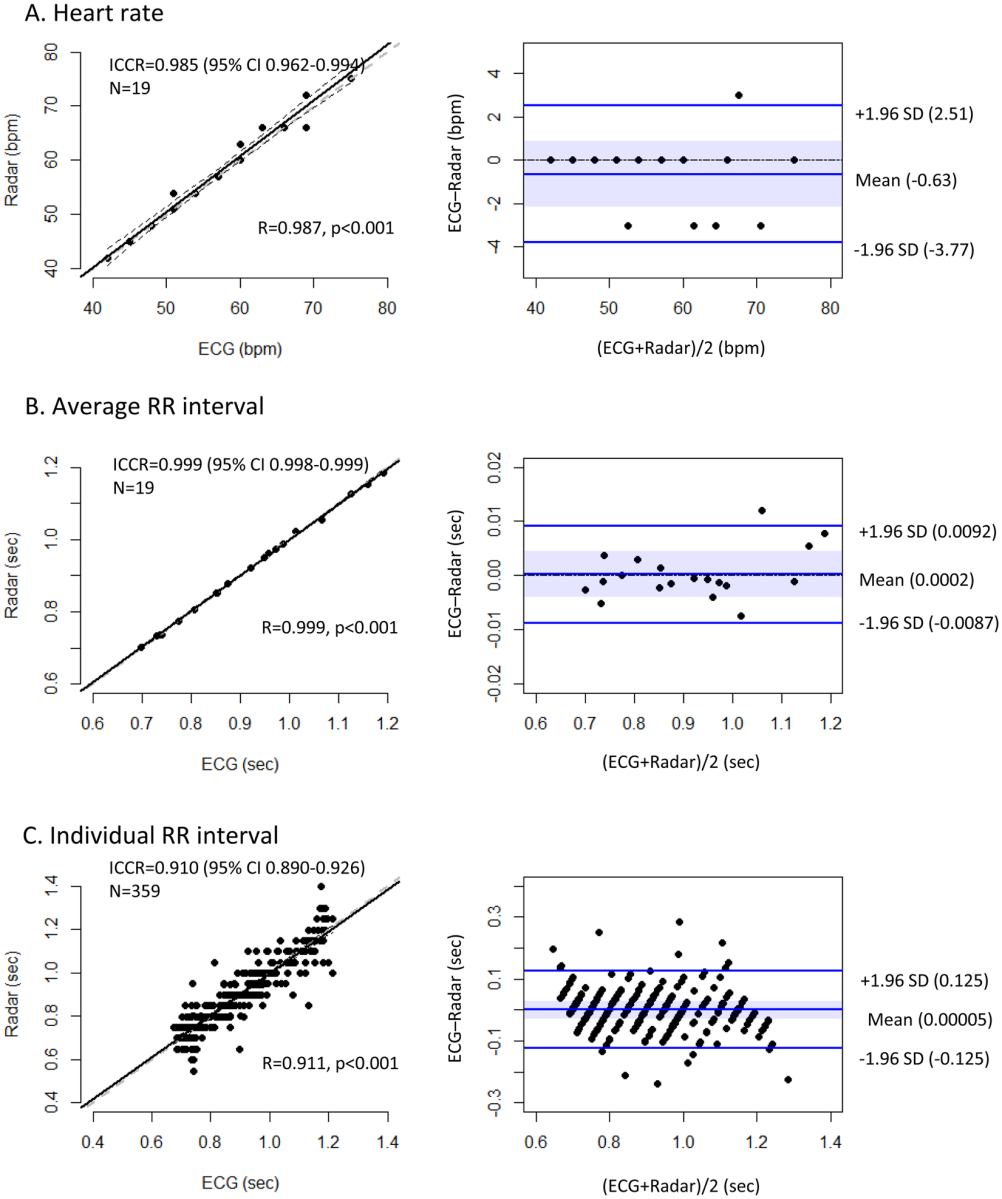
Agreement between the radar and ECG in the NSR group. The PR (**A**), average R-R interval (**B**) and the individual R-R intervals (**C**) were measured in the NSR group using IR-UWB radar and ECG. There was excellent agreement between the two methods for both the PR and average R-R interval. The individual R-R intervals measured using radar also agreed well with those measured using ECG, although the agreement level was slightly lower than that for the PR and average R-R interval. ^†^ECG, electrocardiography; ICCR, intraclass correlation coefficient.

In the NSR group, the PR and average R-R interval measured using radar showed excellent agreement with those measured using ECG. The BA plots showed no significant biases between the PR and average R-R interval measurements obtained using the two methods. The agreement level of the individual R-R interval was slightly lower than that of the average R-R interval between the two measurement methods, but it was still very high. The BA plot showed no significant bias between the measurements of individual R-R intervals obtained from the two methods (Fig. 6).

**Figure 7:**
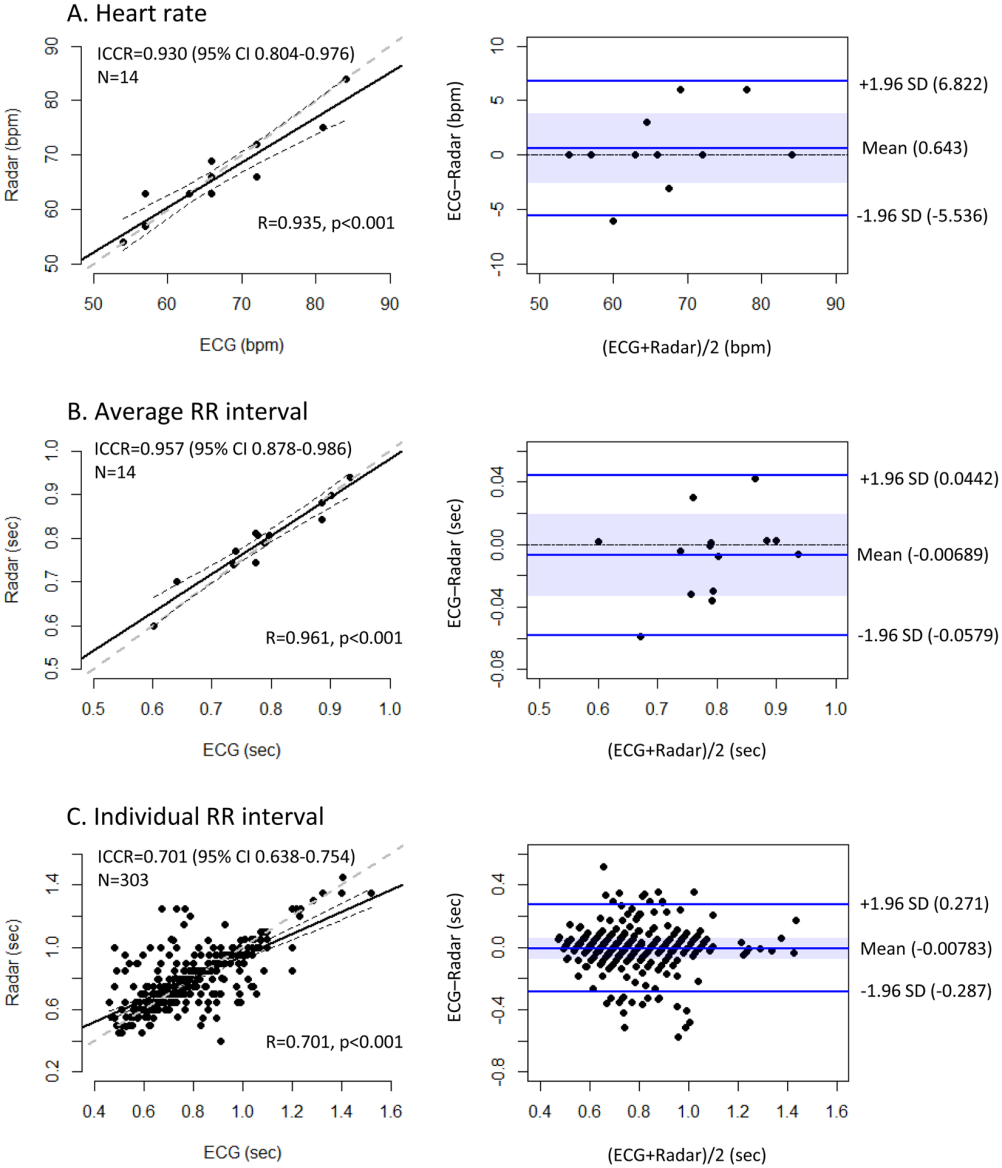
Agreement between radar and ECG in the PeAF group. The PR (**A**), average R-R interval (**B**) and individual R-R intervals (**C**) were measured in the PeAF group using IR-UWB radar and ECG. Both the PR and average R-R interval showed strong agreement between radar and ECG. The level of agreement for the individual R-R intervals between the two methods was significantly lower than that for the PR and average R-R intervals but was still sufficiently high. ^†^ECG, electrocardiography; ICCR, intraclass correlation coefficient.

The levels of agreement between the radar and ECG measurements of the PR, average R-R interval and individual R-R interval were lower in the PeAF group than in the NSR group. However, the agreement between the two methods for the PR and average R-R interval was still excellent. The agreement level between the two methods for the individual R-R intervals was lower than that for the average R-R intervals but was still sufficiently high. The BA plot showed no significant biases between the two methods for the PRs, average R-R intervals and individual R-R intervals, although the 95% limit of agreement was wider for the measurements in the PeAF group than those in the NSR group (Fig. 7).

**Figure 8:**
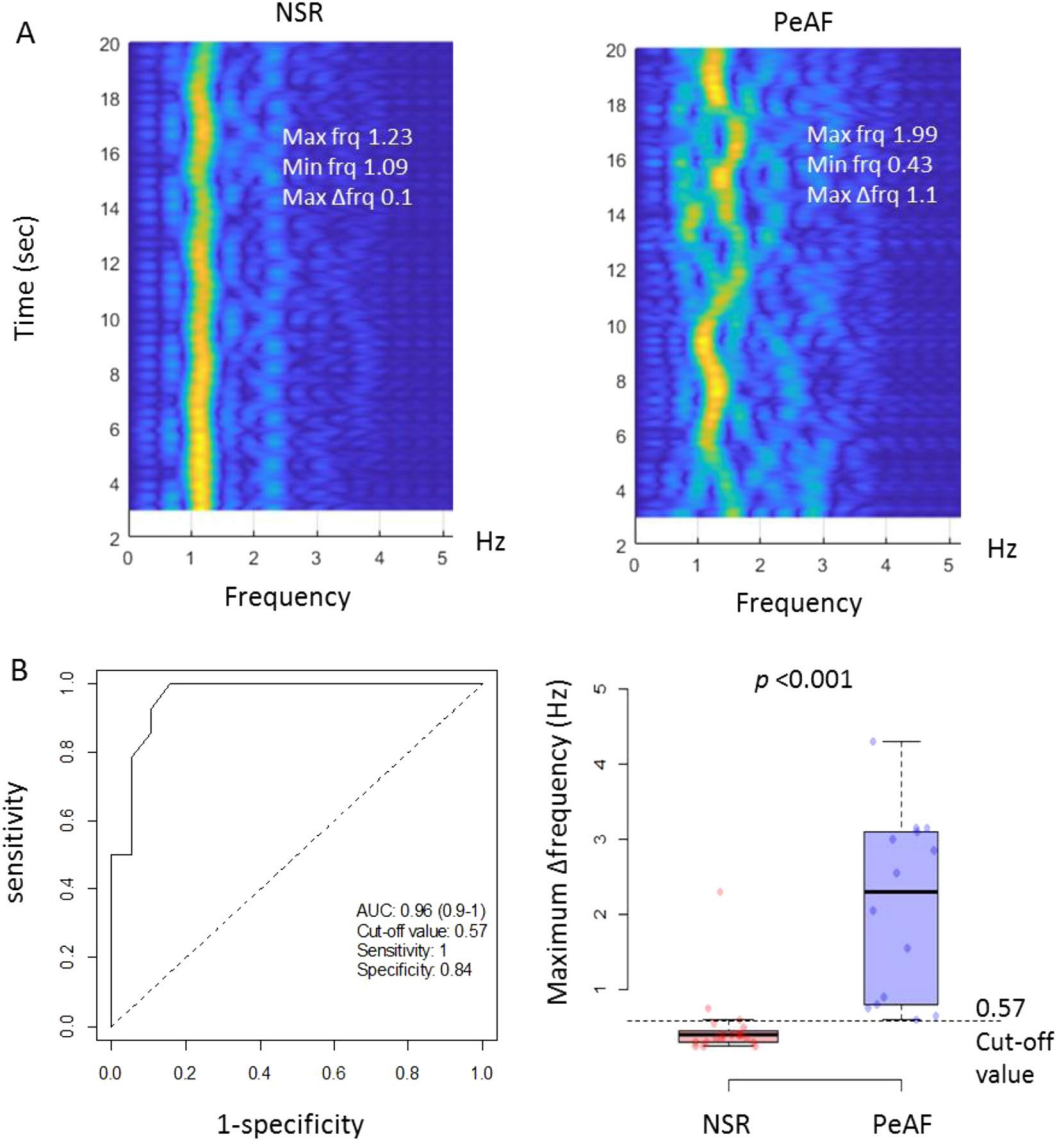
Differentiation between NSR and PeAF by the analysis of the frequency domain. The frequencies of the radar signal intensities were color-coded and plotted against time on 2-dimensional spectrograms (**A**). The brighter-colored line represents the frequency at the peak signal intensity. The Max∆frq appeared to be greater in the patients with PeAF (1.1 Hz) than in those with NSR (0.1 Hz). In the receiver operating characteristic curve analysis, the Max∆frq showed very high diagnostic performance for PeAF at a threshold of 0.57 Hz (**B**). NSR, normal sinus rhythm; PeAF, persistent atrial fibrillation; Max, maximal; Min, minimal; frq, frequency; Max∆frq, maximum frequency variation in the peak signal intensity.

To detect PeAF automatically using IR-UWB radar, the frequency domains of the radar signals was analyzed. The two distinguishing spectrograms obtained from representative patients with NSR or PeAF are depicted in Fig. 7. The frequency of the peak signal intensity varied little over time in the patients with NSR, whereas the frequency widely shifted during the measurement period in the patients with PeAF (Fig. 8A). The MaxΔfrq of the radar signals was greater in the PeAF group than in the NSR group (median [interquartile range] of 2.3 [0.83–3.08] Hz vs. 0.40 [0.30–0.45] Hz, respectively; *p* < 0.001). The receiver operating characteristic curve analysis showed a C-statistic of 0.96 (95% CI, 0.90–1.00), a sensitivity of 1.00 and a specificity of 0.84 at the 0.57 Hz threshold set at the maximum value of Youden’s J-point (Fig. 8B and Table 2). The high correct classification rate (0.909) and Cohen’s Kappa (0.819; 95% CI 0.627–1.000) indicated an excellent level of agreement between the classifications of the rhythms using radar and those using ECG at the threshold level (Table 2).

**Table 2:** Accuracy of the IR-UWB radar in detecting PeAF.

		ECG	
		PeAF (N = 14)	NSR (N = 19)
Radar	PeAF (N = 17)	14	3
Max∆frq 0.57 Hz	NSR (N = 16)	0	16

Sensitivity = 1.00, specificity = 0.84, CCR = 0.909.

Cohen’s Kappa = 0.819 (95% CI 0.627–1.000).

## Discussion

In this study, we found that respiration and carotid pulsation could be simultaneously monitored from the neck in a noncontact manner and under normal breathing conditions using IR-UWB radar. The RR, PR and average R-R interval measured using radar agreed excellently with those measured using manual counting and ECG in both patients with NSR and patients with PeAF. Individual R-R intervals measured using radar and ECG also agreed well, although the agreement levels were slightly lower than those of the average R-R intervals in the both patient groups. We also demonstrated that PeAF could be accurately differentiated from NSR using the MaxΔfrq of the carotid radar signals.

Conventional vital sign monitoring methods, including ECG, pulse oximetry based on photoplethysmography and impedance pneumography require direct contact with the patient’s skin, thus causing limited patient mobility, discomfort and the risk of skin injuries and contagious diseases^4–6^. To date, several noncontact vital sign monitoring methods have been introduced, including pressure sensors, capacitors, photoplethysmography devices using a high-definition digital camera and ambient light, thermography, laser Doppler vibrometers and continuous-wave Doppler radar^18–22^. However, few of these methods have been validated in clinical settings, and none of them have demonstrated feasibility and accuracy sufficient for use in daily clinical practice.

Radar measures the movement of an object at a distance using a band of radio waves in the electromagnetic spectrum. Because of its ultra-wide bandwidth, IR-UWB radar can provide high resolution and good penetration power, enabling it to recognize the fine motions of a human body, including respiratory activity and heartbeat, with only >0.01% of the energy emitted from a usual 5-GHz WiFi transmitter^7–9^. This very low energy emission guarantees the long-term safety of IR-UWB radar and makes it suitable for international approval by multiple agencies^23^. Because radar employs radio waves, its measurements are not influenced by skin color, ambient light conditions or weather, in contrast with photoplethysmography^24^. The ultra-wide bandwidth also enables radar to have a simple structure with a small antenna, making its installation and operation more feasible than that of other noncontact vital sign monitors. The high penetration power of IR-UWB radar provides a further advantage over photoplatysmography and laser vibrocardiography in that clothing or blankets will not interfere with the radar measurements^25^. In these regards, IR-UWB radar is a good candidate for a noncontact continuous vital sign monitoring technology.

In our previous study^17^, we introduced a novel noncontact heartbeat monitoring technique utilizing IR-UWB radar and demonstrated the high accuracy of the resulting HR measurements. During the study, the radar measurements were directly obtained from the anterior chest of the patients. However, the obvious problem in the study was the measurement condition in which the patients were required to hold their breath to minimize respiratory chest wall motions, which would make it almost impossible for the technique to be used in clinical applications. In the current study, we have overcome this obstacle by relocating the measurement site to the neck. We discovered that we could even measure the tiny cyclic movements of the carotid artery during the systolic and diastolic phases of the heart using IR-UWB radar. Compared with radar measurement from the anterior chest, measurement from the carotid artery resulted in significantly reduced signal input from respiratory activity which could then be effectively suppressed by the algorithm while calibrated simultaneously with the carotid pulse. Through this modification, we could accurately measure the HR and R-R interval from carotid pulses, simultaneously with the RR, under normal breathing conditions. This change in the measurement condition is an important improvement that enables IR-UWB radar to be utilized for continuous vital sign monitoring for a prolonged period in stationary patients. This improvement could expand the potential clinical applications of IR-UWB radar to include vital sign monitoring for intensive care units and for patients with highly contagious diseases.

It is also an important development that we measured the pulse waveform in the carotid artery using IR-UWB radar. Located just beneath the skin of the neck, the carotid artery is one of the largest arteries in the human body. Because of its shallow location relative to the body surface, tiny movements of the arterial wall caused by cyclic pressure differences inside the carotid artery can be detected on the skin of the neck using sensitive devices. De Melis *et al*.^26^ have also introduced a noncontact method for the measurement of carotid artery pulse using laser Doppler vibrocardiography. Given that we have already accurately measured individual heartbeats represented by R-R intervals in the anterior chest using IR-UWB radar in our previous study^17^, accurate measurement of individual R-R intervals from the carotid artery may open the possibility for noncontact measurement of the pulse transit time using radar. The pulse transit time contains information associated with the stiffness of the artery and has already been widely exploited to assess the degree of atherosclerosis and vascular aging^27,28^. Therefore, accurate measurement of the carotid pulse would widely expand the applications of IR-UWB radar in the clinical cardiovascular field.

Our results show that IR-UWB radar could accurately differentiate between NSR and PeAF using the MaxΔfrq. In the previous study^17^, we accurately distinguished PeAF from NSR using two methods: analysis of the distribution of individual R-R intervals and analysis of the MaxΔfrq in the power-spectral density relation. Because the signals from the carotid pulse should continuously flow from the subject, and arrhythmia should be detected in real time in this study, we decided to use the MaxΔfrq, which is the simpler of the two approaches. The diagnostic performance (sensitivity of 1.00 and specificity of 0.83) of the MaxΔfrq for PeAF and the agreement level (Cohen’s *K* = 0.87) in the diagnosis of PeAF between radar and ECG reported in our previous study were surprisingly similar to those obtained in this study (1.00, 0.84 and 0.82, respectively)^17^. Although we did not validate our results externally in this study, the high diagnostic performances similar to those in the previous study increase our confidence in the MaxΔfrq as an indicator of PeAF. Real-time detection of AF may be useful in cardiovascular intensive care units, given that the sudden development of AF often indicates the deterioration of patients with cardiovascular diseases and a worsening prognosis^29^.

This study has several limitations. First, although we modified our measurement methods to eliminate the necessity of breath-holding during signal acquisition, the radar sensor remained sensitive to the subject’s motions and could not completely differentiate random motions from respiratory activity and carotid pulses. Therefore, patients undergoing radar measurements should minimize the movement of their upper bodies, except for breathing, during the measurements. However, the accuracies of contact vital sign monitoring methods, including ECG, pulse oximetry and impedance pneumography, are also easily influenced by the subject’s movements. Moreover, we are developing next-stage signal processing algorithms that could rapidly recalibrate the best signal location automatically after a movement. Second, the results are only valid within experimental settings and for the subject posture used in this study. The results may vary with the distance and angle of the radar device in relation to the subject’s neck. There might also be other experimental settings under which IR-UWB radar could extract the carotid pulse better than the settings used in this study. To apply IR-UWB radar in daily clinical practice, its performance in measuring the carotid pulse should be evaluated in various postures and directions. Third, the rhythm classification algorithm using the MaxΔfrq occasionally misclassified NSR as PeAF, whereas it classified PeAF perfectly. The same result occurred in our previous study. We think that this misclassification occurred not due to flaws in the algorithm, but rather because not all NSRs have completely regular R-R intervals. Our current algorithm only considers the MaxΔfrq eventually corresponding to the individual R-R intervals measured by ECG. Using this approach, NSRs with variable R-R intervals are simply impossible for any advanced algorithm to differentiate. Recognizing movements in the cardiovascular system corresponding to atrial motions using radar would be the ultimate solution.

## Conclusion

Using IR-UWB radar, the RR, PR, R-R intervals and heart rhythm could be simultaneously measured with high precision from the necks of normally breathing subjects. Moving the measurement site to the neck enabled us to measure the carotid artery pulse accurately using radar, in the presence of signals from respiratory activity. This novel approach using an alternative measurement site could expand the applications of IR-UWB radar as a noncontact vital sign monitoring technology from the current short-term detection of the HR and rhythm to the prolonged monitoring of the RR, PR and rhythm. Soon, carotid pulse waveforms obtained using IR-UWB radar could facilitate further additional applications of radar in cardiovascular clinical practice.

### Supplementary information

Supplementary Table 1 and 2
